# Strategies to enhance resilience to cope with workplace adversities post-COVID-19 among ICU nurses

**DOI:** 10.4102/curationis.v45i1.2345

**Published:** 2022-10-24

**Authors:** Nombulelo E. Zenani, Gopolang Gause, Leepile Sehularo

**Affiliations:** 1 Quality in Nursing and Midwifery (NuMIQ) Focus Area, Faculty of Health Sciences, School of Nursing Science, North-West University, Mmabatho, South Africa

**Keywords:** strategies, intensive care unit nurses, resilience, coping, COVID-19

## Abstract

**Background:**

It is critical for intensive care unit (ICU) nurses to develop resilient coping strategies to cope with workplace adversities. The coping strategies will mitigate the development of maladaptive psychological disorders prone to working in a stressful environment.

**Objectives:**

The aim of this study is to analyse previous literature conducted on strategies that enhance resilience in ICU nurses to cope with workplace adversities beyond the coronavirus disease 2019 (COVID-19) pandemic. The study was conducted by examining all available global literature in the context of the aim of the study.

**Method:**

An integrative literature review was chosen for the study. Purposive sampling method was used to select the relevant databases to answer the review question, namely Google Scholar, EBSCOhost, Medline and Nursing/Academic Edition. The search terms used were ‘strategies’, ‘resilience’, ‘intensive care unit nurses’, ‘coping’, ‘workplace adversities’, ‘beyond COVID-19’ and post ‘COVID-19’.

**Results:**

Three themes emerged from the study, namely promoting personal attributes, effective relational support and active psychological support.

**Conclusion:**

Enhancing resilience among ICU nurses requires both intentional individualised care from the ICU nurses and a systematic approach by nursing management that will meet the psychological needs of ICU nurses when working in a stressful ICU environment.

**Contribution:**

The findings of the review have highlighted specific strategies of improving resilience in ICU nurses, which can ultimately create a safe working environment in the ICU.

## Introduction

In December 2019, the coronavirus 2019 (COVID-19) pandemic arose and globally all nurses working in the intensive care unit (ICU) were most likely to be exposed to the COVID-19 virus from the patients who were diagnosed with a respiratory infection (Peñacoba et al. 2021). This placed the ICU nurses at a greater risk of contracting the virus and spreading it to the public and their families, which influence the daily development of fear and guilt among the ICU nurses (Peñacoba et al. 2021). At the origin of the pandemic, the ICUs became congested within a few weeks, which led to a huge need in increasing the upscaling of the ICU beds and additional resources to treat the patients (Liu et al. 2020). This phenomenon led to an increase in labour resources and enhancement of infection control practices (Liu et al. 2020; Muller et al. 2020; Peñacoba et al. 2021).

The catastrophic health service emergency alongside the high transmission rate of COVID-19 rate caused significant psychological distress among the ICU nurses (Muller et al. 2020). The ICU nurses dealt with consistent traumatic patient experiences and unexpected loss of families, friends and colleagues, which led to several mental health problems for the ICU nurses (Muller et al. 2020). These challenges lead to ICU nurses experiencing fear, which influenced their daily activities such as sleep and rest (Fernandez et al. 2021). Fear of the unknown dominated the ICU nurses and ICU management when new knowledge kept evolving how they operate in their role. A study that looked at the experiences of ICU nurses during the COVID-19 pandemic by Moore et al. (2022) in the United States revealed that ICU nurses felt unsafe and feared for their lives while providing care for the COVID-19 infected patients. They were uncertain if the N95 masks are a sufficient infection and prevention measure, as the pandemic progressed there was an exacerbation of limited personal protective equipment (PPE), which increased the feeling of being unsafe and putting their lives at risk. In addition to their frustration, knowledge as to how COVID-19 is transmitted and the disease process and treatment was questioned, which led to changes in infection control policies, treatment and nursing care measures in the ICU (Moore et al. 2022). A cross-sectional study conducted in China showed that in the first wave of COVID-19 there were about 32% of ICU nurses had direct exposure to COVID-19 (Chegini et al. 2021). Furthermore, the exposure to COVID-19 illuminated fear, anxiety, and depression among ICU nurses (Chegini et al. 2021). Furthermore, literature has shown that some ICU nurses have suffered physically from the impact of the COVID-19 pandemic experiencing chronic headaches, insomnia and psychological symptoms, which in severe cases some contemplated suicidal thoughts (Levi & Moss 2022; Shen et al. 2020; Tan et al. 2020). Moreover, the ICU nurses were challenged ethically, as the ICU obligated the nurses to provide patients with the best individualised care. However, because of the increasing number of ICU patients, the nurses were unable to provide the best care, in instances of end-of-life care, they were unable to providing closure to the families by nursing the patients in the presence of their loved one’s because of COVID-19 restriction visiting in the ICU units. This resulted in a sense of guilt and hopelessness among the ICU nurses and this took a toll on ICU nurses’ ethical standing (Chegini et al. 2021; Hossain & Clatty 2022; Moore et al. 2022). Moreover, the distraction of the clinical practice, the loss of sense of control and the subsequent fear of the destabilisation of healthcare services provoked conditions such as post-traumatic stress disorder (PTSD) and generalised anxiety disorders among ICU nurses (Rahman et al. 2020).

The ICU environment as per norm is a specialised advanced care setting, which is prone to create much economical and psychological pressure because of the high cost of care and the sensitivity of the critical care patients in need of advanced technological care (Mealer et al. 2012). The environment exposes the ICU nurses to challenges related to workplace stress, which results from prolonged exposure to working with patients who are in a life-threatening state (Mealer et al. 2012). The ICU environment exposes the nurses to perform cardiovascular resuscitation, prolonging life with artificial support, dealing with anxious patients’ families and the multidisciplinary team demands (Rahman et al. 2020). Moreover, the workplace stress within the ICU is influenced by the high turnover rates of ICU nurses, which directly increases the healthcare cost and limits quality care because of an imperfect number of nurses in the ICU (Mealer et al. 2012; Stenbrook 2002). The ICU nurses have no other choice but to adapt to the environment and the demands that come with working in the ICU. The most critical coping mechanism utilised by the ICU nurses is resilience (Da Silva & Barbosa 2021).

Resilience is a multidimensional characteristic that embodies the personal quality that enables the ICU nurses to thrive during the workplace adversities (Mealer et al. 2012:294). Mealer et al. (2012) defined resilience as the ability of the ICU nurses to cope with their work environment and be able to maintain a healthy and stable psychological functioning, despite the exposure to extreme stressors. According to Huffman et al. (2021), the characteristics of a resilient nurse include being able to observe stressors to overcome challenges, having optimism, embracing commitment and being adaptive to change (Huffman et al. 2021). Furthermore, the same authors argue that resilient ICU nurses have a significantly low prevalence of experiencing burnout and PTSD (Huffman et al. 2021). In the ICU, resilience assists in adjustment following trauma from persistent exposure to resuscitation, nursing a dying patient and sudden death of a recovering patient from the sudden change in the disease process (Mealer et al. 2021). Lee et al. (2015) explained that enhancing resilience among ICU nurses will provide the nurses with the courage to cope with stress, it will promote a positive working environment and the cost of burnout can be decreased. Furthermore, the intervention of promoting resilience in ICU nurses assists in the loss of hours from absenteeism and improves retention of ICU nurses (Lee et al. 2015).

Despite the given discussion, there seems to be a scarcity of literature on this phenomenon. Therefore, this integrative literature review will provide an in-depth understanding of what are the best strategies through a review of literature, regarding the strategies that can enhance the resilience of ICU nurses to cope with workplace adversities. The results will contribute to the body of knowledge in nursing education and clinical practice on how to promote sound working environments for ICU nurses post COVID-19. This study will provide recommendations on what strategies can be considered to enhance resilience in ICU nurses, to cope with workplace calamity. Based on the this discussion, the following review question was asked by the researchers ‘what are the strategies that can enhance the resilience of ICU nurses to cope with workplace adversities beyond the COVID-19 pandemic?’

## Aim of the study

The aim of this study is to analyse previous literature conducted on strategies that enhance resilience in ICU nurses to cope with workplace adversities beyond the COVID-19 pandemic.

## Design and method

This study employed an integrative review methodology. According to Whittemore and Knafl (2005), an integrative literature review is a scientific review method that summarises previous empirical and theoretical data to provide a broad understanding of a particular phenomenon or healthcare concern. The review consists of five stages, namely identifying the problem, literature search, data evaluation, data analysis and the presentation of the study (Whittemore & Knafl 2005). The integrative literature was seen best for the study, as it allowed the authors to have a primary lens in synthesising various streams of literature to generate new knowledge on strategies that can be adopted to enhance resilience in ICU nurses to cope with workplace adversities beyond the COVID-19 pandemic (Whittemore & Knafl 2005).

### Problem identification

The first stage of the integrative literature review is to have a clear identification of the problem and purpose that the review will be addressing (Whittemore & Knafl 2005). Literature reveals that during the COVID-19 pandemic, the nursing personnel was experiencing formidable stress in the ICU. The major problems leading to stress and other mental health problems are extensive workload, long-term fatigue and repetitive exposure to critical and dying patients (Levi et al. 2021; Şanlıtürk et al. 2021; Shen et al et al. 2020). Therefore, it is critical to provide measures that aid to prevent further psychological effects, especially after the impact of COVID-19 on the psychological well-being of ICU nurses. The ICU nurses should be availed of an environment where there are less severe psychological stressors that impair their role and they are able to cope amidst the normal work adversities within the ICU. Thus, the purpose of this study is to analyse previous literature conducted on strategies that enhance resilience in ICU nurses to cope with workplace adversities beyond the COVID-19 pandemic.

### Literature search

A well-constructed and defined literature search strategy is vital for enhancing the rigour in an integrative literature review mainly because unclear, incomplete and biased searches result in inaccurate results (Whittemore & Knalf 2005). A comprehensive search in integrative research is suitable to identify a maximum number of eligible primary sources (Whittemore & Knalf 2005). Therefore, a systematic search process was used to locate the relevant literature. The inclusion criteria consisted of qualitative and quantitative research and literature reviews on resilience strategies for ICU nurses. The articles included were only written in English, which is a language understood by all authors. Studies written in non-English language and studies that relate to resilience outside the ICU setting such as resilience for undergraduate nursing students or non-healthcare practitioners were excluded. This ensured that the study focused on achieving the relevant articles that address the enhancement of resilience in ICU nurses to cope with workplace adversities beyond the COVID-19 pandemic.

The literature search process involved searching three key databases, namely Google Scholar, EBSCOhost, Medline and Nursing/Academic Edition. The search was from articles from 2019 to 2022, which was during the commencement of the COVID-19 pandemic and posts for broader knowledge regarding resilience within the ICU for nurses. The search strategy used the following keywords: ‘strategies’, ‘resilience’, ‘ICU’, ‘nurses’ and COVID-19. The search was based on the following PICO scheme:

- **Population**: Studies conducted on ICU nurses
- **Intervention**: Strategies to enhance resilience
- **Comparison**: No comparison
- **Outcome**: Increased resilience of ICU nurses

**FIGURE 1:**
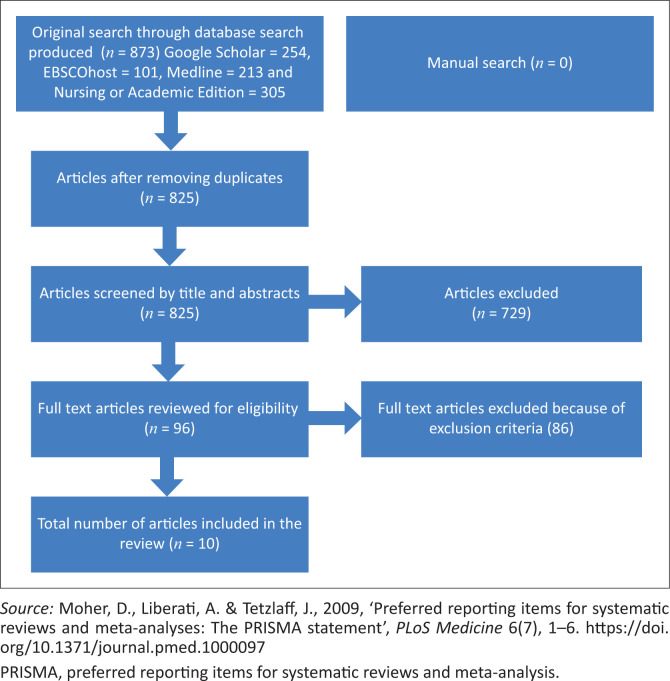
PRISMA flow chart of the search strategy.

The relevant sources were selected; data were extracted and then analysed and synthesised to answer the review question, ‘What are the strategies that can enhance the resilience of intensive care unit nurses to cope with workplace adversities beyond the COVID-19 pandemic?’. The first two authors read the article’s title alongside the abstracts of the identified sources from the search. This was followed by conducting a screening of the obtained articles from the search. A further selection of the articles was performed by analysing the full context of the articles to ensure they meet the PICO scheme of the study. Both researchers agreed on the inclusion of the articles and disagreements were resolved by the involvement of the third author (see Figure 1).

### Data evaluation

In this stage, the extraction of specific methodological features of the obtained studies is recommended in order to evaluate the overall quality of the study (Whittemore & Knafl 2005). In this study, all the included authors were involved in the data evaluation stage to limit errors during the data extraction process. The authors used a data extraction tool in the form of a matrix to facilitate the process of critical appraisal (Brink et al. 2020). The appraisal of the methodological strength of the study and the limitations was conducted using John Hopkins Nursing Evidence-based Practice Research Evidence Appraisal tool as described by Newhouse et al. (2007). All the authors used the quality appraisal tool to grade the evidence in the study and exclude studies with low quality that will not be able to assist with answering the aim of the review. The grades included using High quality, Good quality, Low quality or Major flaws. The quality of the selected articles was based on the author’s surname, the year of the study, the aim, design, sample and the results of the study (see Table 1).

**TABLE 1:** A detailed description of the included studies†.

Author(s) and year	Aim	Design and sample	Rigour
Alkhawaldeh et al. 2020	To summarise and appraise the methodological quality of primary studies on interventions for the management of occupational stress among intensive and critical care nurses	Systematic review, 2330 studies yielded, 69 duplicates and excluded and 2248 excluded after screening and 12 studies met the inclusion criteria	The aims and objectives are clear. Design is relevant. Results are consistent. Implications are discussed. *Quality Appraisal = High Quality (A).*
Brown & Abuatig 2020	To explore the concept of resilience and how resilience training for nurses can help protect against the negative effects of stress caused by organisational change	Literature review, 11 articles met the inclusion criteria	The aims and objectives are clear. Design is relevant. Results are consistent. Implications are discussed. *Quality Appraisal = High Quality (A).*
Cadge et al. 2021	To explore and describe how nurses experience providing care for patients hospitalised with COVID-19 in ICUs.	Qualitative study, professional nurses (*n* = 16)	The aims and objectives are clear. Design is relevant. Results are consistent. Implications are discussed. *Quality Appraisal = High Quality (A).*
Huffman et al. 2020	To explore and describe the well-being of healthcare professionals during the COVID-19 pandemic	A quantitative study, using a 42-item survey examining specific stressors, grit and resilience was widely distributed to physicians, residents, fellows and administrators at a large academic institution for departmental distribution (*n* = 785).	The aims and objectives are clear. Design is relevant. Results are consistent. Implications are discussed. *Quality Appraisal = High Quality (A).*
Mealer et al. 2021	Pilot and in-person or virtual mindfulness-based cognitive therapy programmes enhance resilience among critical nurses	Randomised trial, ICU nurses (*n* = 54)	The aims and objectives are clear. Design is relevant. Results are consistent. Implications are discussed. *Quality Appraisal = High Quality (A).*
Muller et al. 2020	To assess and summarise available research on the mental health impact of the COVID-19 pandemic on healthcare workers, including (1) changes over time, (2) prevalence of mental health problems and risk/resilience factors, (3) strategies and resources used by healthcare providers to protect their own mental health, (4) perceived need and preferences for interventions and (5) healthcare workers’ understanding of their own mental health during the pandemic	Rapid systematic review, 59 studies included	The aims and objectives are clear. Design is relevant. Results are consistent. Implications are discussed. *Quality Appraisal = High Quality (A).*
Owen & Schimmels 2020	To explore and describe the application of psychological first aid principles in guiding nursing leadership during dynamic times	Literature review, nine articles included	The aims and objectives are clear. Design is relevant. Results are consistent. Implications are discussed. *Quality Appraisal = High Quality (A).*
Shanefelt et al. 2020	To explore sources of anxiety among healthcare professionals during COVID-19	Qualitative, focus interviews with ICU nurses and physicians and (*n* = 69)	The aims and objectives are clear. Design is relevant. Results are consistent. Implications are discussed. *Quality Appraisal = High Quality (A).*
Wei et al. 2020	To determine the perceptions of self-care strategies to combat professional burnout among nurses and physicians in paediatric critical care units.	Qualitative, descriptive study with a phenomenological overtone. Intensive care unit nurses and physicians (*n* = 20)	The aims and objectives are clear. Design is relevant. Results are consistent. Implications are discussed. *Quality Appraisal = High Quality (A).*
Yu et al. 2020	To assess ICU nurse’s resilience and identify associated personal and physical activity behaviours using a job demand-recovery framework	Quantitative study, online survey. Intensive care unit nurses (*n* = 103)	The aims and objectives are clear. Design is relevant. Results are consistent. Implications are discussed. *Quality Appraisal = High Quality (A)*

*Source*: Adapted from Kangasniemi, M., Pakkanen, P. & Korhonen, A., 2015, ‘Professional ethics in nursing: An integrative review’, *Journal of Advanced Nursing* 71(8), 1744–1757. https://doi.org/10.1111/jan.12619

ICU, intensive care unit; COVID-19, coronavirus disease 2019.

† , A - high quality; B - good quality; C - low quality.

### Data analysis

The data analysis stage during the integrative review requires that the data should be ordered, coded, categorised and summarised into a unified and integrated conclusion about the research problem (Whittemore & Knafl 2005). In this stage, the authors read all the obtained articles to understand the context against the research aim of the study, which is to analyse previous literature conducted on strategies that enhance resilience in ICU nurses to cope with workplace adversities beyond the COVID-19 pandemic.

The three authors of the study analysed the 10 selected articles independently using thematic data analysis. The key terms that emerged from the articles were highlighted, summarised and listed. After which, they were categorised according to themes and subthemes. The findings are displayed in Table 2 as three main themes and each with subtheme. In the integrative literature review method, the data analysis stage consists of data reduction, data display, data comparison, conclusion and verification (Whittemore & Knafl 2005). In the data reduction phase, the authors divided the sources into subgroups to facilitate the analysis (Whittemore & Knafl 2005) (see Table 2).

**TABLE 2:** Details of the data reduction.

Authors and year	Article title	Findings	Context/setting
Alkhawaldeh et al. 2020	Stress management interventions for intensive and critical care nurses: A systematic review	The 12 studies included discussed that condition cognitive training and mindfulness-based interventions are effective in managing workplace stress among ICU nurses.	Global literature
Brown & Abuatig 2020	Resilience as a strategy to survive organisational change.	Training healthcare professionals on resilience benefits them both professionally and personally by enhancing their self-awareness, confidence and assertiveness.	Global literature
Cadge et al. 2021	Intensive care unit nurses living through COVID-19: A qualitative study	Nursing leadership in ICU needs to be evaluating crisis models that will balance the needs of the patients and that of the nursing personnel.	Boston, United States
Huffman et al. 2020	How resilient is your team? Exploring healthcare providers’ well-being during the COVID-19 pandemic	Higher resilience was associated with lower stress, anxiety, fatigue and sleep disturbances.	Unites states
Mealer et al. 2021	The presence of resilience is associated with a healthier psychological profile in ICU nurses: Results of a national survey	The presence of high resilience in these nurses was significantly associated with a lower prevalence of post-traumatic stress disorder, symptoms of anxiety or depression, and burnout syndrome (< 0.001 for all comparisons).	United States
Muller et al. 2020	The mental health impact of the COVID-19 pandemic on healthcare workers, and interventions to help them: A rapid systematic review	The COVID-19 led to the increased prevalence of PTSD and general anxiety disorders among healthcare workers. Therefore, mental healthcare interventions offered to ICU nurses should be individualised in approach and proactive to make it less stigmatising and more effective.	Global literature
Owen and Schimmels 2020	The application of psychological first aid	Nurse leaders have a great potential to digress to counterproductive capacity during crisis and high stress. Their prompt and active leadership can aid in the morale and psychological well-being of those they lead.	Global literature
Shanefelt et al. 2020	Understanding and addressing sources of anxiety among healthcare professionals during the COVID-19 pandemic	Healthcare professionals need to be supported with substantial psychological support, based on their request and from healthcare professionals’ evaluation. This will in return limit anxiety they experience while functioning in their scope under stressful situations.	Stanford, California
Wei et al. 2020	Self-care strategies to combat burnout among paediatric critical care nurses and physicians	It is important for healthcare professionals to take care of themselves to best take care of others. Promoting self-care enhance professional accountability and reduce risk of burnout.	United States
Yu et al. 2020	Physical activity and personal factors associated with nurse resilience in ICU	Nurses with high resilience levels have a greater tolerance to work in a highly physical environment.	Auckland, New Zealand

ICU, intensive care unit; PTSD, post-traumatic stress disorder; COVID-19, coronavirus disease 2019.

### Presentation

According to Whittemore and Knafl (2005), integrative literature reviews can be reported in the form of a table. The details from the primary sources should be explicit with supporting evidence for a logical chain of evidence. In alignment with Whittemore and Knafl (2005), the authors provided a table with the results, indicating the themes and subthemes and discussed the findings and the limitations of the study.

### Ethical considerations

No permission was needed to conduct the study. However, to maintain research integrity the authors acknowledged the authors of all the articles whose study’s were used in the writing of this article in the in-text and list of references.

## Results

Three themes with each having sub-themes emerged from the study. Table 3 shows the themes and sub-themes that emerged from the study.

**TABLE 3:** Themes and subthemes.

Themes	Sub-themes
1. Promoting personal attributes	1.1 Emotional recharge
2. Effective relational support	2.1 Peer support 2.2 Promote open communication
3. Active psychological support	3.1 Mindfulness-based cognitive behavioural therapy 3.2 Managing workplace stress 3.3 Training on resilience

### Strategy 1: Promoting personal attributes

Promoting personal attributes was the first theme to be identified in this integrative review. One of the sub-themes that emerged for the first strategy was performing emotional recharge. According to Wei et al. (2020), activities that include self-reflecting, praying, spending time with family and friends, having a clear balance and boundaries between home and work, getting sufficient sleep and moderate exercise aid as emotional recharge. These activities assist ICU nurses to develop a positive attitude in dealing with adverse work experiences and help to achieve a healthy psychological well-being. Thus, enhancing their resilience. The same authors also advocate for ICU nurses to find meaning in their work as a form of inner compass. It is the inner compass/higher purpose knowledge about the ICU nurses’ role that will guide ICU nurses’ actions when on duty. The authors further elaborate to say that the acknowledgement of the higher purpose in the ICU role will also positively influence mindset and motivate the ICU nurses to do their day-to-day tasks with passion and love. This action further enhances resilience when the nurses encounter challenges in the ICU (Wei et al. 2020). Yu et al. (2020) supported the findings and stated that regular exercise can enhance the level of resilience and thus improve ICU nurse’s well-being. The same author emphasises that the promotion of rest and relaxation techniques can attenuate negative outcomes of job demands in ICU such as chronic back pains, stress and burnout (Yu et al. 2020).

### Strategy 2: Effective relational support

The second theme identified in this review is effective relational support. Sub-themes emerged are peer support and promoting open communication. According to Owen and Schimmels (2020), in enhancing resilience among ICU nurses, peer support that is facilitated by experienced colleagues is essential. Peer support is an evidence-based intervention, which reduces distress and builds resilience after a stressful traumatic event in the ICU unit (Owen et al. 2021). The authors further elaborated that peer support through social connection and emotional support promotes a sense of safety, calmness and builds community, which instils hope for recovery. Cadge et al. (2021) in a qualitative study with ICU nurses in America concur with the given findings. In this study, the nurses reported that turning to each other for support, regardless of the available employee assistance programme was more practical and effective in enhancing their resilience to cope with workplace adversities. The ICU nurses felt more comfortable and safe talking to peers who had shared similar experiences (Cadge et al. 2021). Cadge et al. (2021) recommended open communication from ICU unit managers through pre-shift huddles, check-ins and updates on new information through texts and emails as great support especially to new nurses deployed in ICU units for the first time. Owen and Schimmels (2020) recommended the use of eight core actions that can enhance the work environment to support building of resilience among ICU nurses. The first action is communication or consistent engagement by ICU leaders. The communication establishes human connection through validating the ICU nurses’ feelings and thoughts. This action provides ICU leaders an opportunity to view the ICU nurses distinctively as individuals, respond uniquely and enforce the best decisions to be made at different situations in the ICU units that meet the needs of the ICU nurses (Owen & Schimmels 2020).

### Strategy 3: Active psychological support

The third theme that emerged from this integrative literature review is active psychological support. The sub-themes include mindfulness cognitive behaviour therapy, managing workplace stress and training on resilience. From the reviewed literature, three authors recommended mindfulness-based cognitive therapy (MBCT) as means of active psychological support when ICU nurses are experiencing psychological implications from the workplace adversities and stress (Huffman et al. 2021; Mealer 2020; Shanefelt et al. 2020). According to Mealer et al. (2021), MBCT reduces stress, has great benefits in assisting ICU nurses to be aware of negative feelings and thoughts and further teaches the ICU nurses to think differently about troubling workplace stress experiences, which further enhances resilience. The secondary sub-theme that emerged was managing workplace stress. Alkhawaldeh et al. (2020) affirmed that ICU leaders can adopt stress management interventions to reduce the severity of workplace stress that can lead to serious mental health problems. The authors stated techniques and programmes such as mindfulness-based intervention, massage and yoga. The techniques increase the ICU nurses’ ability to cope with stress and thereby safeguard the ICU resources (Alkhawaldeh et al. 2020:46). The last sub-theme that materialised from the sources was the importance of resilience training for ICU nurses. According to Huffman et al. (2020), a feasible 12-week training developed by Mealer (2020) for ICU nurses, which covered skills such as self-care, cognitive therapy, expressive writing and event-triggered counselling sessions enhanced the resilience of ICU nurses. Brown and Abuatiq (2020) supported the previous author and indicated that resilience training amid ICU nurses enhances resilience, reduces stress and promotes the well-being of ICU nurses. The training benefits the ICU nurses both professionally and personally by enhancing self-awareness, assertiveness and self-care, which ultimately assist when working in the ICU stressful environment (Brown & Abuatiq 2020; Mealer 2020).

## Discussion

Nurses who work in the ICU are at a high risk to suffer from work-related occupational stress as a result of organisational factors within the ICU unit (Babanataj et al. 2019). This is related to factors such as high turnover, conflicts with the ICU team, feeling of inadequacy, burnout and extensive work demands from their role and management (Babanataj et al. 2019; Mealer 2020). During COVID-19 the workplace adversities intensified as more patients were being admitted in ICU and occupational stress mounted up among the ICU nurse (Cadge et al. 2021; Lie et al. 2020; Peñacoba et al. 2021). Based on those findings, it is evident that ICU nurses’ psychological well-being needs to be improved through exploring strategies that can enhance their work efficiency in the ICU while working with the patients. The study has revealed that improving resilience through self-care is vital; it assists the ICU nurses to develop a positive attitude in dealing with adverse situations and achieve an optimal psychological well-being (Wei et al. 2020). As nurses, it is easy to lose oneself at the duty of care by consistently working to meet the needs of the patients and compromising one’s physical, emotional, social and psychological health in the process. However, the study has revealed that investing in ‘emotional hygiene’ through praying, socialising, exercising, etc., provides the ICU nurses with enough strength and willingness to provide quality care to the patients (Wei et al. 2020; Yu et al. 2020).

This study has further revealed that resilience plays a significant role in mitigating burnout and preventing mental health disorders (Wei et al. 2020). Biologically, uncontrolled stressors affect the brain cognitive functioning, which leads to compromise with logical thinking and judgement of the ICU nurse (Wei et al. 2020). Seeking social support and practising mindfulness according to the study results helps to improve brain function such as working memory, actively coping amidst stressful situations and assist to regulate emotions effectively (Cadge et al. 2021; Wei et al. 2020). The results of the study have revealed that social support can increase work relations among the ICU nurses, which in turn enhance resilience for the nurses to cope with the ICU stressful environment (Cadge et al. 2021). Therefore, the role of having a sound relationship with colleagues is vital. These relationships create a safe space for communicating fears and concerns, which assist the ICU nurse to not feel isolated or stressed (Cadge et al. 2021).

Furthermore, the study has revealed that prompt referral for mindfulness cognitive therapy is vital to ICU nurses who present with mental health disorder symptoms. The active and prompt response assists the ICU nurses to reduce stress and enables them to regulate their emotions and thoughts when dealing with stressful situations, which enhance resilience (Huffman et al. 2021; Mealer et al. 2021; Shanafelt, Ripp & Trockel 2020). The study has revealed a critical need for resilience training as a strategy to enhance resilience among the ICU nurses. The knowledge and the skills that the ICU nurses will learn from the intervention will bring awareness to what is resilience, make them aware if they still have resilience and how they can develop the skill timeously (Brown & Abuatiq 2020; Huffman et al. 2020; Miller et al. 2020).

### Limitations

This research focused mainly in the context of ICU nurses and environment, and it is not necessary for all healthcare professionals. Therefore, the findings cannot be generalised for other healthcare disciplines.

### Recommendations

To promote a healthy working environment in the ICU, strategies that enhance resilience are critical to enforce a safe working space, especially for ICU nurses. A strong determinant of satisfaction in the ICU units is justice and sound support. Managers within the ICU can enhance the resilience of ICU nurses by demonstrating fair practice and optimisation on policies and procedures that strengthen good communication and distribution of new information to the ICU nurses. From this study, it is evident that peer support counselling is successful in providing psychological assistance to the ICU nurses. Thus, the ICU leaders can advocate for the selection of suitable peer supporters to be trained on effective interpersonal skills and counselling skills required to support the ICU nurses during and after ICU traumatic events. Further studies can be conducted to investigate the effects of mindfulness in resilience to combat burnout in the ICU. Further studies can also be conducted to investigate the impact of resilience training for ICU to mitigate job satisfaction in ICU, especially within the African countries as there were less studies in that context.

## Conclusion

This study achieved its aim to analyse previous literature conducted on strategies that enhance resilience in ICU nurses to cope with workplace adversities beyond the COVID-19 pandemic.

The review highlighted the importance of the intentional balance of workplace lifestyle from the ICU nurses and a systematic approach to intervention from the ICU management, which includes clear communication, psychological support and availability of training on resilience. Both these approaches enhance resilience and set a therapeutic working environment for the ICU nurses to cope with workplace adversities.
